# Ball tipped guide wire for broken nail removal: a case report

**DOI:** 10.1051/sicotj/2021010

**Published:** 2021-03-08

**Authors:** Faizan Iqbal, Mehroze Zamir, Nasir Ahmed, Syed Wajahat Kamal, Nouman Memon

**Affiliations:** Department of Orthopaedic Surgery, Patel Hospital 75300 Karachi Pakistan

**Keywords:** Femur fracture, Intramedullary nail, Union, Broken nail, Guidewire

## Abstract

A broken intramedullary nail is a well-known complication of non-union of femur shaft fractures. Numerous surgical techniques have been presented before in patients with non-union of long bone fractures. We report the surgical technique used to perform removal of the broken distal segment of a nail in a patient who achieved uneventful union after intramedullary nailing of closed femur shaft fracture. A ball-tipped guidewire was inserted through the broken segment of the femur nail. A pre-bend plain wire was then inserted. With the help of a vise-grip, both wires were twisted in order to make a secure handle between guidewires and a broken implant. With the help of a mallet upward-directed blows were applied to extract a broken segment of the nail. We found ball-tipped guidewire technique a useful and effective technique in removing the broken distal portion of the nail.

## Introduction

A broken intramedullary nail is a well-known complication after non-union of long bone fractures. On other hand, the broken intramedullary nail is not frequently encountered once the union has been achieved [1]. Several techniques have been described previously for extracting a distal broken portion of nail especially after non-union [2, 3]. Extracting a broken distal portion of the nail is a challenging surgery even for experienced surgeons [4]. Therefore surgeon must be aware of techniques of extracting the broken distal portion of the nail and must keep all required instruments available during the removal of broken hardware. In this article, we described our simple ball-tipped guide-wire technique of removing a distal broken portion of the nail which was not amenable to remove with the nail extracting techniques described previously. A full written and informed consent was taken about the submission of the concerned case for publication.

## Case report

**Figure 1:**
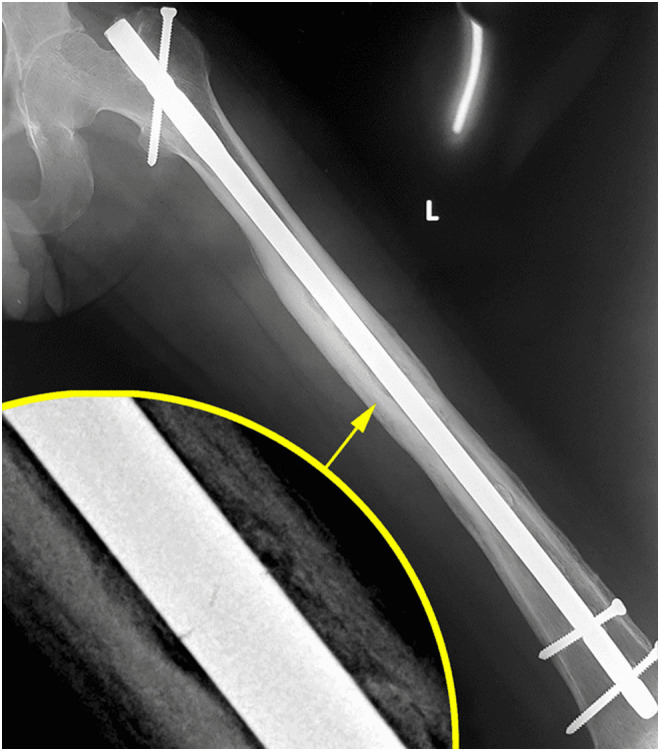
Antero-posterior view of full-length X-ray of the left femur showing broken femur nail with complete bony union.

**Figure 2:**
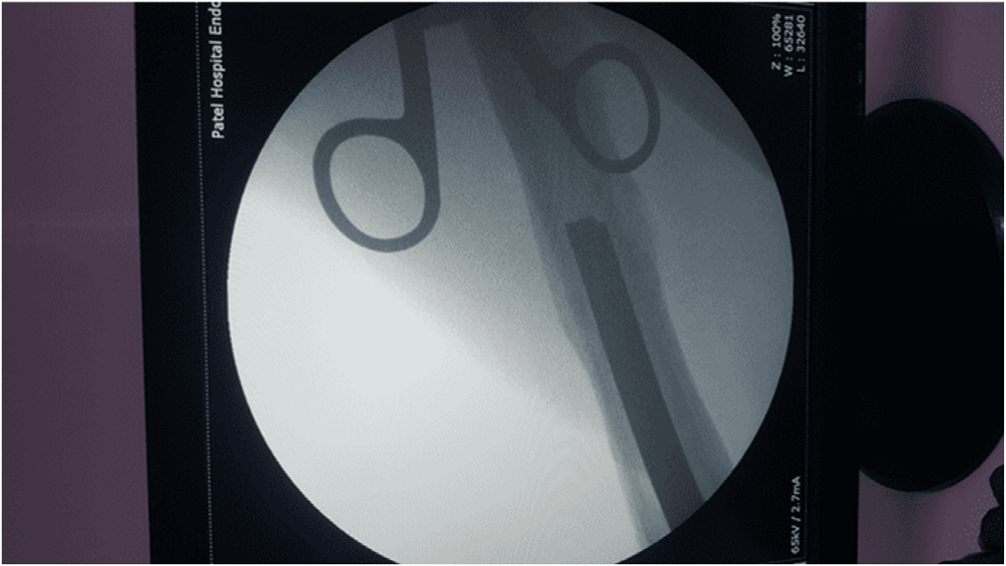
Proximal portion of the nail removed with distal broken portion of nail remains in situ.

**Figure 3:**
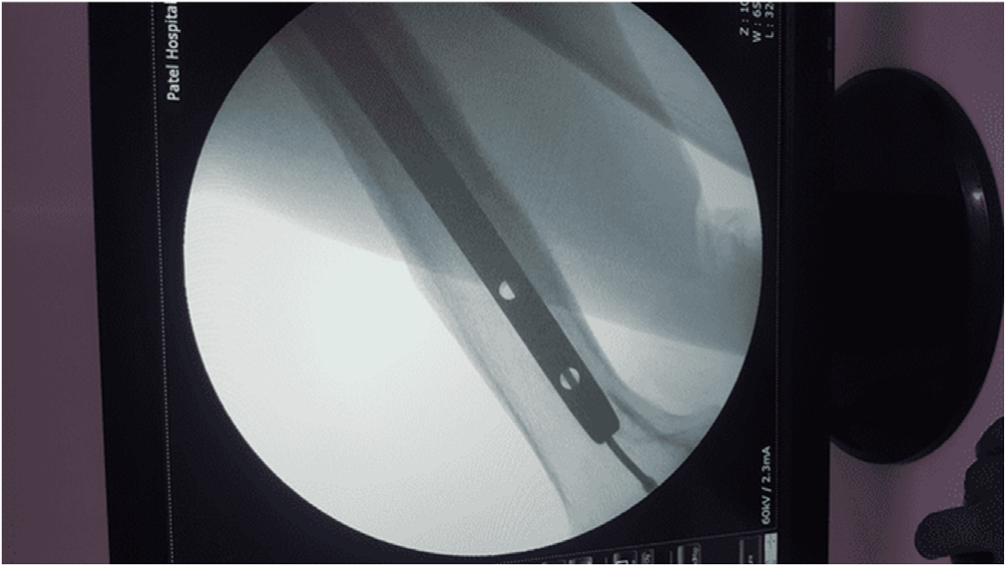
Ball tipped guidewire inserted through a broken segment of the nail.

**Figure 4:**
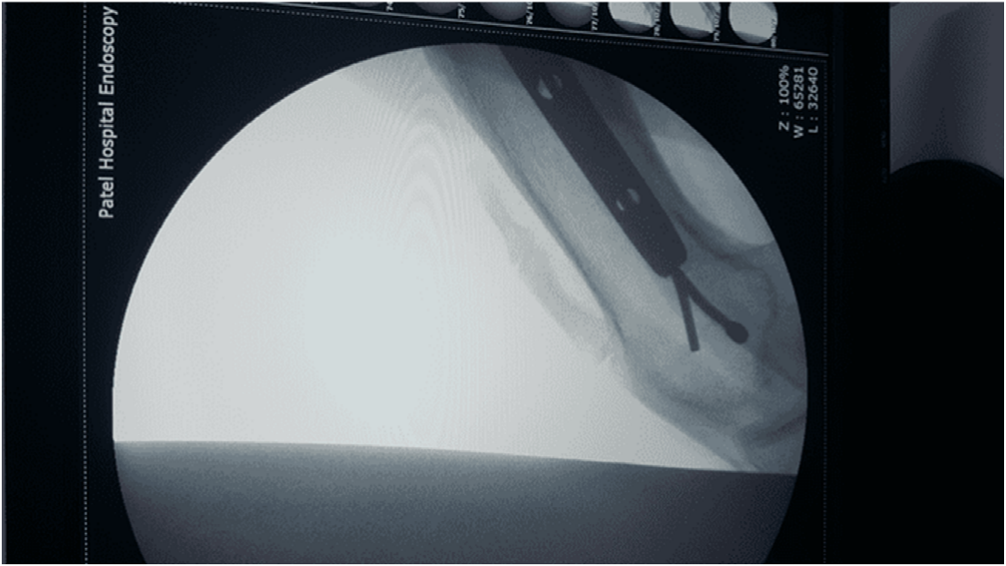
Both wires (Plain & Ball tipped) protruded through the broken portion of the nail.

**Figure 5:**
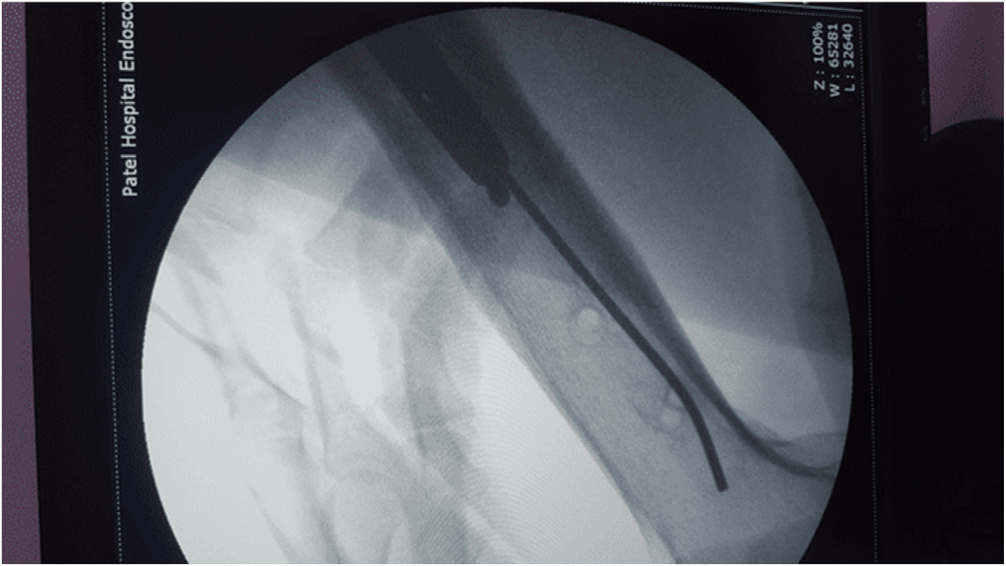
Extraction of the broken nail with plain guide wire remains in situ.

**Figure 6:**
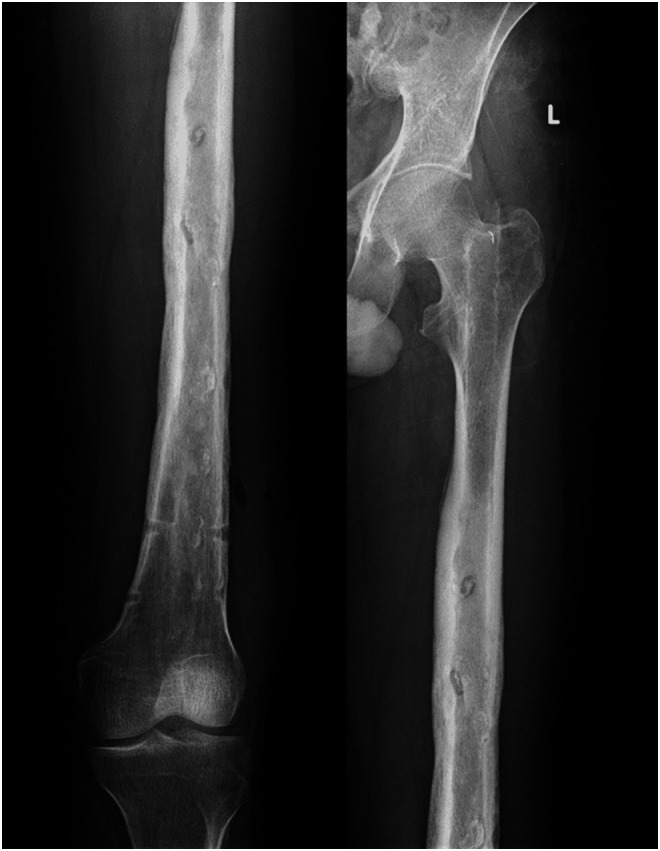
Complete removal of the broken distal segment of the nail.

In 2005, a 33-year-old male experienced a closed left femur mid-shaft fracture after a motor vehicle accident (MVA). The very same day, he underwent for the interlocking nail of the left femur. There was an uneventful postoperative period and the union was achieved after 12 weeks. After 15 years, he presented with pain over the left thigh area primarily localized to the distal screw site with prominent distal-most screw-on local examination. This discomfort was presumed to be due to implant-related, so a decision was made to remove the hardware. Pre-operatively broken distal segment of the nail was not anticipated as it was very difficult to pick that segment radiographically (Figure 1). The device for removing the broken distal portion of the nail was not arranged pre-operatively. The patient was laterally placed and the previous incision was utilized to extract the nail. Before removing the proximal and distal locks, a universal jig for nail removal was tightened initially. After extracting the proximal and distal locks, a mallet was used to extract the nail. It soon becomes clear that only the proximal portion of the nail has come out and the remaining distal portion of the nail remains in situ (Figure 2). A long hook tool that was originally built to retrieve the broken part of the nail is not readily available in the operation room, so alternate plans have been quickly implemented. Initially, an effort was made to remove a broken nail portion with a pre-bend ball-tipped wire, but ultimately failed. Then a ball-tipped wire was threaded through the broken nail to engage the distal portion of the nail (Figure 3). In order to ensure optimum interference fit between two guidewires, a pre-bend simple guidewire was then threaded through a broken nail segment (Figure 4). Vise-grip was used to proximally hold two wires together and then a mallet was used to apply continuous upward force by direct blows on ball-tipped guidewire to retain plain guidewire in situ (Figure 5). Sadly, this effort failed as well. In order to make a secure handle between guide wires and broken implant, both wires were also twisted with the help of vise-grip, and then mallet was used to apply upward guided force by direct strikes, leading to the extraction of the distal broken portion of the nail, which was then verified in the radiograph intensifier (Figure 6).

## Discussion

Removal of broken hardware is a challenging surgery even for an experienced trauma surgeon. Therefore, the surgeon must be aware of the simple and efficient techniques for removing broken hardware. A ball-tipped guide-wire technique is a simple and efficient technique for extracting a broken distal portion of nail and can be performed in a situation where it is not easy to access special equipment for extracting broken nail. Before our surgical technique, various methods for removing broken nail segments have also been implemented (Table 1). Most techniques involve special instruments to extract a distal broken portion of the nail that might not actually be present at the time of surgery, especially in times when it was difficult to anticipate broken segment of the nail on radiographs pre-operatively.

**Table 1:** Techniques of removing broken nail.

Study	Technique	Comments
Metikala and Mohammed [5]	Ball tipped wire passed through the knee retrograde and nail fragment extracted in antegrade manner	Violation of knee joint
Zhao and Slater [6]	Plain guidewire passed through nail fragment and extracted out distally by a cortical window below the fragment. Flexible reamer then used over the guidewire to push fragment out of the window	Cortical window leads to additional surgical insult to the bone. Refixation will need cables as mentioned by the author. Larger broken fragments and very distal fragments are not amenable to this technique
Pongsamakthai et al. [7]	Insertion and impaction of a T-reamer in the canal of nail fragment followed by antegrade extraction	Appropriately sized T reamer must be available. More suited for slotted nails as impaction may be better. Damage to the reamer and generation of metal particles inside the medullary canal are a possibility
Mazzini et al. [8]	Fracture site exposed and nail fragments extracted via cement rongeur and cement extraction hook	Special instrumentation is necessary. Opening of fracture site is also required

The surgical technique we used in our case is recommended in situations where the union has already been achieved or the fracture site opening is not desirable (e.g., when treating hypertrophic non-unions). Our technique is also effective in cases where special surgical equipment for removing a broken portion of the nail is not readily available in operation theatre [5].

The only limitation of our surgical technique is that it would not be used in a situation when small diameter nails were used to fix long bone fractures. We recommend using this method only when dealing with a nail of the diameter of 10 mm or above that allows easy placement of a ball-tipped wire and one or more plain guide wires. In our technique, cortical windows are not needed to remove distal broken nails, which is an added advantage [6].

Previous literature supports the use of kuntscher nails, small diameter nails, small reamers, and extraction hooks in circumstances where nail diameter is small [7]. The standard technique of removing broken hardware with a hook is not effective due to multiple reasons such as inadequate grip, unable to engage nail tip buried deep into subchondral bone, and failure to remove a large distal broken portion of the nail because of inadequate strength [8]. We were lucky to have a nail diameter large enough to allow both wires (plain and ball-tipped wires) to pass through the nail. In cases when dealing with a large diameter nail; the broken distal portion may be extracted with a long nail extractor with a distal hook, multiple ball-tipped guidewire, or a long Kirschner wire with a curved tip [9, 10].

In order to remove damaged Kuntscher nails, Maini et al. [11] used the Enders nail as an extraction method. While intramedullary nailing in an orthopedic culture has gained attention for long bone fractures. During implant removal, hardware breakage must not be overlooked [12, 13]. In order to achieve a good outcome, surgeons must be aware of easy and efficient procedures. We felt that our mentioned technique is an efficient method to remove broken nail fragments.

## Conclusion

We believe that the ball-tipped guide wire surgical technique is an easy and reliable approach for extracting the broken distal segment of the nail in a situation where special equipment for removing broken hardware is not readily available. This technique also avoids cortical window to prevent complications such as an iatrogenic fracture.

## Conflict of interest

The authors declare no conflict of interest.

## Funding

The authors declare that no funding was involved in this study.
